# Construction and validation of nomograms based on the log odds of positive lymph nodes to predict the prognosis of lung neuroendocrine tumors

**DOI:** 10.3389/fimmu.2022.987881

**Published:** 2022-09-23

**Authors:** Suyu Wang, Juan Wei, Yibin Guo, Qiumeng Xu, Xin Lv, Yue Yu, Meiyun Liu

**Affiliations:** ^1^ Department of Anesthesiology, Shanghai Pulmonary Hospital, School of Medicine, Tongji University, Shanghai, China; ^2^ Department of Thoracic Surgery, Shanghai Pulmonary Hospital, School of Medicine, Tongji University, Shanghai, China; ^3^ Department of Cardiothoracic Surgery, Changzheng Hospital, Naval Medical University, Shanghai, China; ^4^ Department of Health Statistics, Naval Medical University, Shanghai, China; ^5^ Department of Orthopaedics, Changzheng Hospital, Naval Medical University, Shanghai, China

**Keywords:** lung neuroendocrine tumor, log odds of positive lymph nodes, predictor, survival, nomogram

## Abstract

**Background:**

This research aimed to investigate the predictive performance of log odds of positive lymph nodes (LODDS) for the long-term prognosis of patients with node-positive lung neuroendocrine tumors (LNETs).

**Methods:**

We collected 506 eligible patients with resected N1/N2 classification LNETs from the Surveillance, Epidemiology, and End Results (SEER) database between 2004 and 2015. The study cohort was split into derivation cohort (n=300) and external validation cohort (n=206) based on different geographic regions. Nomograms were constructed based on the derivation cohort and validated using the external validation cohort to predict the 1-, 3-, and 5-year cancer-specific survival (CSS) and overall survival (OS) of patients with LNETs. The accuracy and clinical practicability of nomograms were tested by Harrell’s concordance index (C-index), integrated discrimination improvement (IDI), net reclassification improvement (NRI), calibration plots, and decision curve analyses.

**Results:**

The Cox proportional-hazards model showed the high LODDS group (-0.79≤LODDS) had significantly higher mortality compared to those in the low LODDS group (LODDS<-0.79) for both CSS and OS. In addition, age at diagnosis, sex, histotype, type of surgery, radiotherapy, and chemotherapy were also chosen as predictors in Cox regression analyses using stepwise Akaike information criterion method and included in the nomograms. The values of C-index, NRI, and IDI proved that the established nomograms were better than the conventional eighth edition of the TNM staging system. The calibration plots for predictions of the 1-, 3-, and 5-year CSS/OS were in excellent agreement. Decision curve analyses showed that the nomograms had value in terms of clinical application.

**Conclusions:**

We created visualized nomograms for CSS and OS of LNET patients, facilitating clinicians to bring individually tailored risk assessment and therapy.

## Introduction

Lung neuroendocrine tumors (LNETs) originate from pulmonary neuroendocrine cells, accounting for approximately 25% of primary lung neoplasms (1). The incidence of LNET is less than 0.002% as reported in some countries (2–4). Owing to the increased lung cancer screening, the annual incidence of LNET has substantially increased, rising from 0.0003% in 1973 to 0.0014% in 2004 in the United States (5–7). Currently, the 2015 World Health Organization (WHO) classification has grouped LNETs into four histologic variants based on their histopathologic features: typical carcinoid (TC), atypical carcinoid (AC), large cell neuroendocrine carcinoma (LCNEC), and small cell lung carcinoma (SCLC) (8). The 5-year survival rate of non-small cell lung cancer (NSCLC) was approximately 16%, while the corresponding rate of SCLC, LCNEC, AC, and TC patients was 5%, 17%, 64%, and 84%, respectively (9, 10). Due to the rarity and morphological heterogeneity of these tumors, there have been limited clinical data available regarding LNETs, thus making their diagnosis, staging, risk assessment, and treatment challenging (1). Although specific to NSCLC, the international American Joint Committee on Cancer/Union for International Cancer Control (AJCC/UICC) tumor-node-metastasis (TNM) staging system has been applied to LNETs (11, 12). However, several studies have shown an overlapping survival of patients with LNETs, particularly in stages II and III (11–15). Therefore, further investigation is warranted to optimize the staging system for LNETs.

Lymph node (LN) involvement is a significant prognostic factor for staging and risk stratification. A combination of the number of positive lymph nodes (NPLN), the number of dissected lymph nodes (NDLN), and the anatomic location of LN metastasis have been applied in the staging of many malignancies (16–20). However, the latest version of AJCC/UICC TNM classification of lung cancer did not take account of any number- or ratio-based LN staging system, which could affect the precision of prognosis evaluation (21, 22). A novel prognostic indicator known as log odds of positive lymph nodes (LODDS) is being utilized to identify patients with a homogeneous prognosis in many malignant tumors, including NSCLC (23–27). However, no specific study has focused on its prognostic significance in LNETs till now.

The present study aimed to determine whether LODDS could be utilized to predict the cancer-specific survival (CSS) or overall survival (OS) of patients with node-positive (N1/N2 classification) LNET using the Surveillance, Epidemiology, and End Results (SEER) database. Based on LODDS, the research intended to construct two visualized and online nomograms which are practical tools for clinical prediction used in many diseases (28–32). To facilitate clinical use, we also constructed two visualized and online nomograms for LNETs.

## Methods

### Study design, data screening, and ethical statement

This is a multi-center retrospective cohort study according to the parts of the methods described in our previous studies (25, 26, 33). We use the Transparent Reporting of a multivariate prediction model for Individual Prediction or Diagnosis (TRIPOD) for reporting (34). The data of this study were downloaded from the SEER 18 registries research database, covering approximately 28% of the population of USA (35). Data were extracted using the SEER*Stat version 8.3.9 software. The requirement for approval by the institutional review board and individual patient consent was waived since the study made use of the database's anonymous data. In summary, this study complied with the Declaration of Helsinki (36).

### Population selection

Data on the patients with lung cancer was obtained from the SEER database. Inclusion criteria were as followed (1): diagnosed from 2004 to 2015; (2) site recode “ICD-O-3/WHO 2008” restricted to “Lung and Bronchus”; and (3) pathologically confirmed as TC (ICD-O-3 code: 8240/3), AC (ICD-O-3 code: 8249/3) or LCNEC (ICD-O-3 code: 8013/3). The study period was set from 2004 to 2015, as the sixth or seventh edition of TMN classification and Collaborative Stage information was available in the database since 2004. Besides, we reclassified the TNM staging system according to the 8th version of TNM classification because the TNM staging system had multiple versions in the SEER database and did not apply to all patients (37). Furthermore, considering its strong invasion ability and unique pathological characteristics limiting the surgical options, SCLC was not included in the present study (38, 39). Although LCNEC was reported to contain subgroups of tumors showing SCLC characteristics and others with NSCLC-like features, surgery could be considered for early and locally advanced LCNEC (40–42). Therefore, we enrolled patients with LCNEC in this study. Patients were excluded who (1) aged<18 years; (2) had a diagnosis of any other cancer; (3) did not undergo radical surgery with systematic LN dissection; (4) had the diagnosis lacking pathological evidence; (5) were at pN0/pN3 disease; (6) had distant metastasis (M1); (7) received preoperative radiotherapy; (8) died within one month after surgery; (9) had unknown information of race, laterality, tumor location, radiotherapy, TNM staging system, and CSS/OS.

### Variable extraction, preparation, grouping, and calculation

The baseline demographics data including age at diagnosis (<65 and ≥65), sex (male and female), and race (white, black, and other) were extracted from the SEER database. Baseline tumor-related characteristics included primary site (upper lobe, middle lobe, lower lobe, and other), laterality (right and left), histotype (TC, AC, and LCNEC), tumor differentiation (well/moderately differentiated, poorly differentiated/undifferentiated, and unknown), T classification (T1, T2, T3, and T4), and N classification (N1 and N2). In addition, treatment information including surgical intervention (sublobectomy, lobectomy, and pneumonectomy), radiotherapy (yes and no/unknown), chemotherapy (yes and no/unknown), NDLN, and NPLN was also extracted from the database. LODDS was calculated as: $LODDS=\text{ln}\frac{NPLN+0.50}{NDLN-NPLN+0.5}$ . To avoid an infinite number, 0.50 was added to both the numerator and denominator. CSS and OS were two of the study endpoints. The period from diagnosis to all-cause death was referred to as OS, while the time from diagnosis to LNET-related death was referred to as CSS. For censored data, the follow-up duration was computed as the number of months between diagnosis and death or the last follow-up (December 31, 2016).

### Construction and validation of nomograms

Baseline features of the study groups stratified by LODDS were compared using Pearson’s χ2 test, Fisher’s exact test, Student t test, or Mann-Whitney test as appropriate. Categorical variables were presented as counts and percentages, while continuous variables were reported as the mean (standard deviation [SD]) or the median (interquartile range [IQR]).

Patients from purchased/referred care delivery areas (PRCDA) of Northern plains, East, and Alaska were considered to be the derivation cohort, whereas the external validation cohort includes patients from PRCDA of Southwest and Pacific coast. LODDS was dichotomized *via* the X-tile software to achieve the largest difference in survival outcome by selecting the highest χ^2^ value in survival analysis indicating the largest survival difference (43). First, age at diagnosis, sex, race, laterality, primary site, histotype, tumor differentiation, T classification, N classification, surgery, radiotherapy, chemotherapy, and LODDS were analyzed by univariable Cox-proportional-hazards regression analysis. Second, the potential predictors with *P*<0.1 were put into the multivariable Cox-proportional-hazards regression analysis using the stepwise Akaike information criterion method (stepAIC) to select the optimal predictors for the final models (44). The results are presented as hazard ratios (HRs) with 95% confidence intervals (CIs). Using these identified prognostic factors, we constructed two nomograms for predicting 1-, 3-, and 5-year CSS and OS in LNET patients.

Several indexes and methods were used to assess the precision of our nomograms. First, Harrell’s concordance index (C-index) was used to evaluate the discrimination power of the nomograms. Second, a calibration plot, a curve presenting the conformity between predicted and actual survival rate at 1, 3, and 5 years, along with bootstrapped sampling of the population, was used to assess the calibration. Third, a comparison between our nomograms and the conventional 8th version of the TNM staging system was conducted by calculating the net reclassification improvement (NRI) and integrated discrimination improvement (IDI) (45). Z test was used to examine the difference. Fourth, the receiver operating characteristic (ROC) curve and decision curve analysis (DCA) were performed to test the clinical usefulness of the nomograms and TNM classification. Kaplan-Maier curves were applied for comparing the discriminative power of nomograms and TNM staging system in the entire study cohort. Finally, to facilitate patients and doctors in using the models, two user-friendly webservers for our nomograms were established.

All statistical analyses were performed using R software (version.3.6.14.1.0; The R Project for Statistical Computing, TX, USA; http://www.r-project.org) and EmpowerStats (version 2.0; http://www.empowerstats.com). Two-tailed *P*<0.05 was deemed as statistical significance.

## Results

### Patients characteristics and cutoff value for LOODS

**Figure 1:**
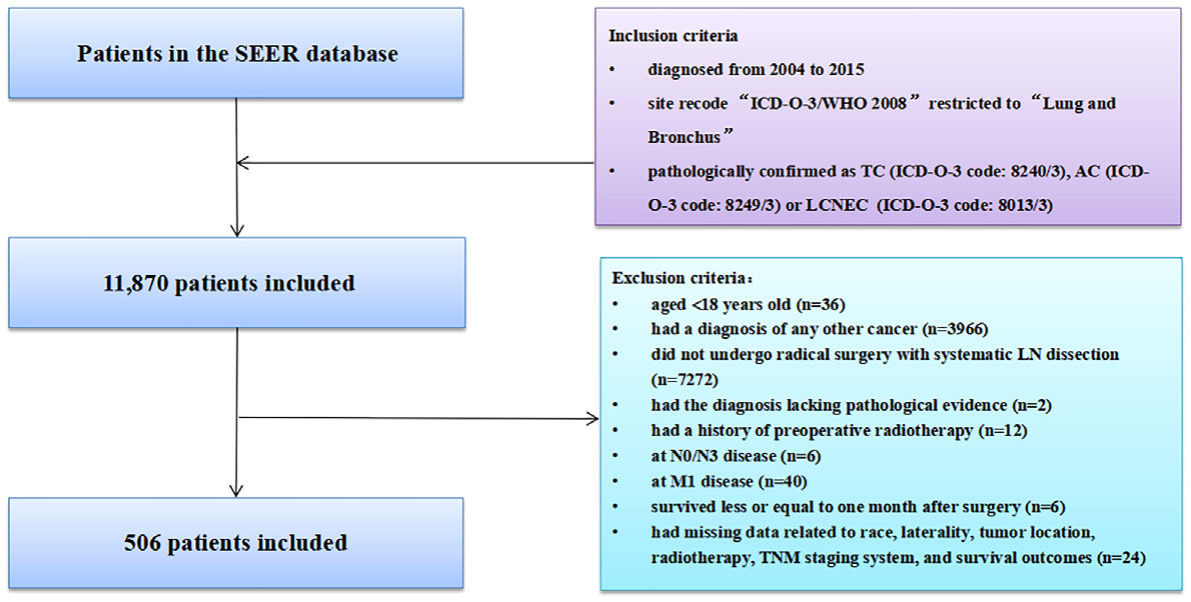
Selection of study cohort from the SEER database. AC, atypical carcinoid; LCNEC, large cell neuroendocrine carcinoma; SCLC, small cell lung carcinoma; SEER, Surveillance, Epidemiology, and End Results; LN, lymph node; TNM, tumor-node-metastasis.

The SEER database collected 11,870 patients diagnosed with LNETs from January 2004 to December 2015. After employing the inclusion and exclusion criteria, 506 patients remained in the final study cohort. The selection process was summarized in **Figure 1**. According to the X-tile software's calculations, the optimal cutoff value of LODDS was set as -0.79. Baseline demographic and clinicopathological variables of participants in the derivation dataset and external validation dataset were summarized in **Table 1**. The median number of NDLN was 9.00 (IQR: 6.00-14.00) in the derivation dataset and 9.00 (IQR: 6.00-15.00) in the validation dataset, whereas the median number of NPLN was 1.00 (IQR: 1.00-2.00) in the derivation dataset and 2.00 (IQR: 1.00-3.00) in the validation dataset. Compared with the derivation dataset, the validation dataset had more patients with other race (*P-*value<0.001). No difference was observed in other variables (all *P-*values>0.05).

**Table 1:** Baseline characteristics of derivation dataset and external validation dataset.

Characteristic	Total (n = 506)	Derivation dataset (n = 300)		External validation dataset (n = 206)	*P* value*
**Age at diagnosis (year)**					0.533
<65	321 (63.44)	187 (62.3)		134 (65.0)	
≥65	185 (36.56)	113 (37.7)		72 (35.0)	
**Sex**					0.314
Male	232 (45.85)	132 (44.00)		100 (48.54)	
Female	274 (54.15)	168 (56.00)		106 (51.46)	
**Race**					<0.001
White	453 (89.53)	281 (93.67)		172 (83.50)	
Black	29 (5.73)	15 (5.00)		14 (6.80)	
Other	24 (4.74)	4 (1.33)		20 (9.71)	
**Laterality**					0.308
Right	281 (55.53)	161 (53.67)		120 (58.25)	
Left	225 (44.47)	139 (46.33)		86 (41.75)	
**Site**					0.360
Upper lobe	194 (38.34)	124 (41.33)		70 (33.98)	
Middle lobe	64 (12.65)	34 (11.33)		30 (14.56)	
Lower lobe	196 (38.74)	113 (37.67)		83 (40.29)	
Other	52 (10.28)	29 (9.67)		23 (11.17)	
**Histotype**					0.100
Large cell neuroendocrine carcinoma	129 (25.49)	86 (28.67)		43 (20.87)	
Carcinoid tumor	274 (54.15)	152 (50.67)		122 (59.22)	
Atypical carcinoid tumor	103 (20.36)	62 (20.67)		41 (19.90)	
**Differentiation**					0.237
Well/moderately differentiated	201 (39.72)	120 (40.00)		81 (39.32)	
Poorly differentiated/undifferentiated	97 (19.17)	64 (21.33)		33 (16.02)	
Unknown	208 (41.11)	116 (38.67)		92 (44.66)	
**T classification**					0.387
T1	209 (41.30)	128 (42.67)		81 (39.32)	
T2	190 (37.55)	114 (38.00)		76 (36.89)	
T3	74 (14.62)	43 (14.33)		31 (15.05)	
T4	33 (6.52)	15 (5.00)		18 (8.74)	
**N classification**					0.468
N1	319 (63.04)	193 (64.33)		126 (61.17)	
N2	187 (36.96)	107 (35.67)		80 (38.83)	
**Surgery**					0.483
Sublobectomy	44 (8.70)	24 (8.00)		20 (9.71)	
Lobectomy	406 (80.24)	246 (82.00)		160 (77.67)	
Pneumonectomy	56 (11.07)	30 (10.00)		26 (12.62)	
**Radiotherapy**					0.733
No/unknown	439 (86.76)	259 (86.33)		180 (87.38)	
Yes	67 (13.24)	41 (13.67)		26 (12.62)	
**Chemotherapy**					0.111
No/unknown	374 (73.91)	214 (71.33)		160 (77.67)	
Yes	132 (26.09)	86 (28.67)		46 (22.33)	
**Number of dissected lymph nodes**	9.00 (6.00-14.75)	9.00 (6.00-14.00)		9.00 (6.00-15.00)	0.080
**Number of positive lymph nodes**	2.00 (1.00-3.00)	1.00 (1.00-2.00)		2.00(1.00-3.00)	0.139
**LODDS as a categorical variable**					0.864
LODDS<-0.79	334 (66.01)	190 (63.3)		132 (64.1)	
LODDS≥-0.79	172 (33.99)	110 (36.7)		74 (35.9)	

Data are listed as median (IQR) or n (%). *P values for Mann-Whitney test or Chi square test by comparing the basic characteristics in the derivation and external validation datasets.

LODDS, log odds of positive lymph node.

### Survival analysis

The median follow-up time for the entire cohort was 42 months (IQR: 19-77 months). Among 506 participants, 180 (35.57%) participants died from any cause, and 135 (26.68%) participants died from LNET-related death in the end of the follow-up. Multivariable Cox regression analysis showed that participants in the high LODDS group (-0.79≤LODDS) was associated with reducing CSS compared to those in the low LODDS group (LODDS<-0.79) (HR=2.21, 95% CI: 1.38-3.52, *P*<0.001) (**Table 2** **)**. The multivariable Cox regression analysis for OS yielded similar results (HR=1.76, 95% CI: 1.20-2.58, P=0.004, **Table 3**). To diminish the potential bias caused by the LN fragments, we performed a sensitivity analysis by restricting the resected LN count to fewer than or equal to 20, and found that LODDS remained statistically significant (CSS: HR=2.06, 95% CI: 1.28-3.31, *P*=0.003; OS: HR=1.68, 95% CI: 1.12-2.53, *P*=0.012) (**Tables S1, S2**). Furthermore, the extent of LN management should be in accordance with the IASLC recommendations, which recommended examination of at least 6 nodes/stations, therefore we excluded patients with the examined LN count to fewer than 6. The multivariable Cox regression analysis yielded similar results (CSS: HR=3.64, 95% CI: 1.99-6.68, *P*<0.001; OS: HR=2.00, 95% CI: 1.23-3.23, *P*=0.005) (**Tables S3, S4**).

**Table 2:** Univariable and stepwise multivariable Cox proportional regression analysis for CSS of the derivation dataset.

Characteristic	N (%)	Univariable analysis			Stepwise multivariable analysis	
		HR (95%CI)	*P* value		HR (95%CI)	*P* value
**Age at diagnosis (year)**						
<65	187 (62.3)	1			1	
≥65	113 (37.7)	2.45 (1.57-3.82)	<0.001		1.82 (1.13-2.93)	0.014
**Sex**						
Male	132 (44.00)	1				
Female	168 (56.00)	0.68 (0.44-1.06)	0.087			
**Race**						
White	281 (93.67)	1				
Black	15 (5.00)	1.03 (0.38-2.81)	0.958			
Other	4 (1.33)	0.00 (0.00-Inf)	0.995			
**Laterality**						
Right	161 (53.67)	1				
Left	139 (46.33)	0.85 (0.54-1.32)	0.467			
**Site**						
Upper lobe	124 (41.33)	1				
Middle lobe	34 (11.33)	0.25 (0.09-0.71)	0.009			
Lower lobe	113 (37.67)	0.59 (0.36-0.96)	0.033			
Other	29 (9.67)	0.41 (0.16-1.04)	0.061			
**Histotype**						
Large cell neuroendocrine carcinoma	86 (28.67)	1			1	
Carcinoid tumor	152 (50.67)	0.08 (0.04-0.14)	<0.001		0.06 (0.03-0.12)	<0.001
Atypical carcinoid tumor	62 (20.67)	0.19 (0.10-0.35)	<0.001		0.16 (0.08-0.31)	<0.001
**Differentiation**						
Well/moderately differentiated	120 (40.00)	1				
Poorly differentiated/undifferentiated	64 (21.33)	6.97 (3.87-12.52)	<0.001			
Unknown	116 (38.67)	1.52 (0.81-2.85)	0.195			
**T classification**						
T1	128 (42.67)	1				
T2	114 (38.00)	0.93 (0.56-1.54)	0.777			
T3	43 (14.33)	1.54 (0.82-2.88)	0.178			
T4	15 (5.00)	0.95 (0.34-2.68)	0.923			
**N classification**						
N1	193 (64.33)	1				
N2	107 (35.67)	1.74 (1.12-2.71)	0.014			
**Surgery**						
Sublobectomy	24 (8.00)	1			1	
Lobectomy	246 (82.00)	0.28 (0.15-0.52)	<0.001		0.69 (0.35-1.34)	0.270
Pneumonectomy	30 (10.00)	0.45 (0.20-1.00)	0.049		1.33 (0.56-3.14)	0.516
**Radiotherapy**						
No/unknown	259 (86.33)	1				
Yes	41 (13.67)	2.34 (1.40-3.89)	0.001			
**Chemotherapy**						
No/unknown	214 (71.33)	1			1	
Yes	86 (28.67)	2.63 (1.69-4.10)	<0.001		0.63 (0.37-1.07)	0.084
**LODDS as a categorical variable**						
LODDS<-0.79	190 (63.3)	1			1	
LODDS≥-0.79	110 (36.7)	2.23 (1.43-3.48)	<0.001		2.21 (1.38-3.52)	<0.001

CSS, cancer-specific survival; LODDS, log odds of positive lymph node; HR, hazard ratio; CI, confidence interval.

**Table 3:** Univariable and stepwise multivariable Cox proportional regression analysis for OS of the derivation dataset.

Characteristic	N (%)	Univariable analysis			Stepwise multivariable analysis	
		HR (95%CI)	*P* value		HR (95%CI)	*P* value
**Age at diagnosis (year)**						
<65	187 (62.3)	1			1	
≥65	113 (37.7)	3.10 (2.12-4.55)	<0.001		2.33 (1.57-3.47)	<0.001
**Sex**						
Male	132 (44.00)	1			1	
Female	168 (56.00)	0.62 (0.43-0.91)	0.014		0.74 (0.51-1.09)	0.131
**Race**						
White	281 (93.67)	1				
Black	15 (5.00)	1.11 (0.49-2.53)	0.801			
Other	4 (1.33)	0.00 (0.00-Inf)	0.994			
**Laterality**						
Right	161 (53.67)	1				
Left	139 (46.33)	0.87 (0.59-1.26)	0.453			
**Site**						
Upper lobe	124 (41.33)	1				
Middle lobe	34 (11.33)	0.41 (0.20-0.87)	0.02			
Lower lobe	113 (37.67)	0.74 (0.49-1.12)	0.149			
Other	29 (9.67)	0.69 (0.35-1.36)	0.284			
**Histotype**						
Large cell neuroendocrine carcinoma	86 (28.67)	1			1	
Carcinoid tumor	152 (50.67)	0.13 (0.08-0.20)	<0.001		0.09 (0.05-0.15)	<0.001
Atypical carcinoid tumor	62 (20.67)	0.23 (0.13-0.39)	<0.001		0.18 (0.10-0.33)	<0.001
**Differentiation**						
Well/moderately differentiated	120 (40.00)	1				
Poorly differentiated/undifferentiated	64 (21.33)	6.22 (3.70-10.46)	<0.001			
Unknown	116 (38.67)	1.98 (1.18-3.34)	0.01			
**T classification**						
T1	128 (42.67)	1				
T2	114 (38.00)	1.12 (0.74-1.70)	0.586			
T3	43 (14.33)	1.35 (0.76-2.40)	0.301			
T4	15 (5.00)	0.91 (0.36-2.31)	0.849			
**N classification**						
N1	193 (64.33)	1				
N2	107 (35.67)	1.76 (1.21-2.57)	0.003			
**Surgery**						
Sublobectomy	24 (8.00)	1				
Lobectomy	246 (82.00)	0.38 (0.21-0.67)	<0.001			
Pneumonectomy	30 (10.00)	0.49 (0.23-1.03)	0.058			
**Radiotherapy**						
No/unknown	259 (86.33)	1			1	
Yes	41 (13.67)	1.98 (1.26-3.12)	0.003		1.51 (0.86-2.66)	0.151
**Chemotherapy**						
No/unknown	214 (71.33)	1			1	
Yes	86 (28.67)	1.86 (1.26-2.73)	0.002		0.43 (0.25-0.74)	0.002
**LODDS as a categorical variable**						
LODDS<-0.79	190 (63.3)	1			1	
LODDS≥-0.79	110 (36.7)	1.71 (1.17-2.48)	0.005		1.76 (1.20-2.58)	0.004

LODDS, log odds of positive lymph node; OS, overall survival; HR, hazard ratio; CI, confidence interval.

### Construction and validation of the nomograms

**Figure 2:**
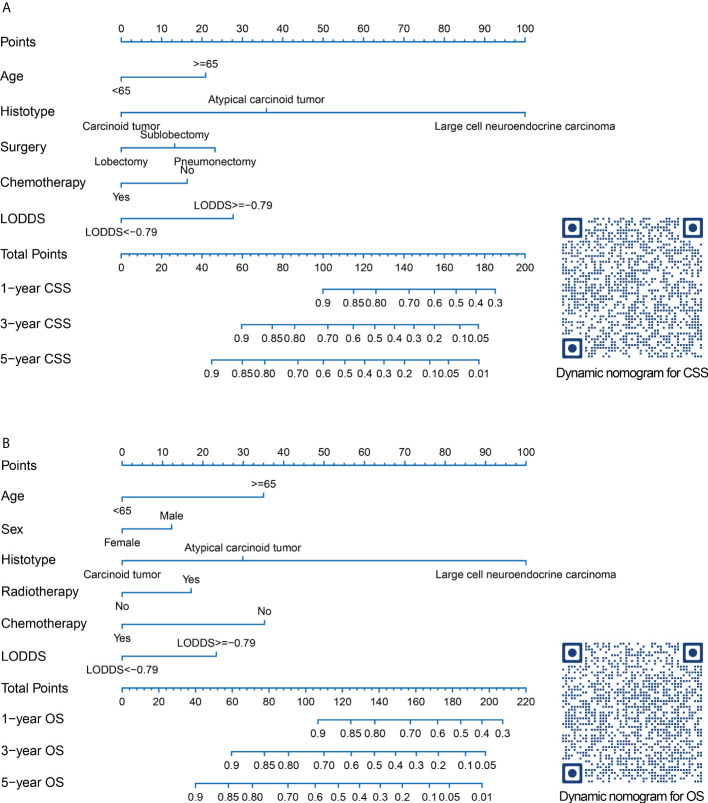
Nomograms and quick response codes of the webservers of the nomograms to predict 1-, 3- and 5-year CSS **(A)** and OS **(B)** for patients with node-positive lung neuroendocrine carcinoma after surgery. CSS, lung cancer-specific survival; OS, overall survival; LODDS, log odds of positive lymph nodes.

Prognostic nomograms for CSS and OS were established including optimal indicators selected by stepAIC method in multivariable Cox regression analysis. For nomogram construction and validation, among the final study cohort including 506 patients, 300 of them were assigned to the derivation cohort (PRCDA=Northern plains, East, and Alaska) and 206 of them were assigned to the validation cohort (PRCDA=Southwest and Pacific coast). The nomogram of CSS showed that histotype made the largest contribution to prediction, followed by LODDS, surgery, age at diagnosis, and chemotherapy (**Figure 2A** and **Table 2**). Similarly, the nomogram of OS showed that histotype made the largest contribution to prediction, followed by chemotherapy, age at diagnosis, LODDS, radiotherapy and sex (**Figure 2B** and **Table 3**). The top point reference scale of the nomograms assigned a score for each category of these predictive variables. After adding up the total score and locating the sum on the total points reference scale, a straight line was drawn to the bottom survival probability scale to find the estimated 1-/3-/5- survival rate. For example, for a LNET patient aged 65 (21 points) who had a diagnosis of large cell neuroendocrine carcinoma (100 points) and underwent lobectomy (0 points) with LODDS<-0.79 (0 points) and received chemotherapy (0 points), the total points were 121 points, corresponding to a 1-year CSS of 83%, in addition, the online dynamic nomogram could give a 95%CI of 74-92% (**Figure 2A**).

**Figure 3:**
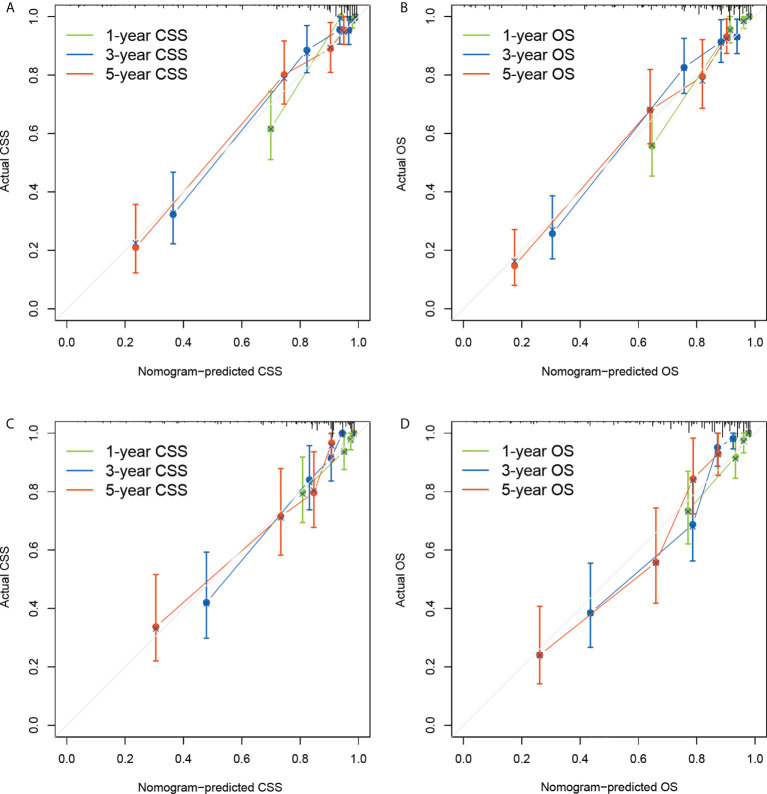
Calibration plots of the nomograms to predict CSS and OS of the derivation dataset **(A, B)** and external validation dataset **(C, D)**. CSS, lung cancer-specific survival; OS, overall survival.

The nomograms were validated internally in the derivation cohort and externally in the validation cohort. C-indexes of nomogram for CSS in derivation cohort and validation cohort were 0.843 (0.801-0.885), and 0.809 (0.755-0.863). C-indexes of OS for OS in derivation cohort and validation cohort were 0.813 (0.774-0.852), and 0.801 (0.753-0.848). Additionally, a good similarity between the nomogram-predicted and actual survival rates was demonstrated by the calibration plots(**Figure 3**), which showed that the spots were near to the 45-degree line.

### Comparison of the nomograms and the eighth edition TNM staging system

**Figure 4:**
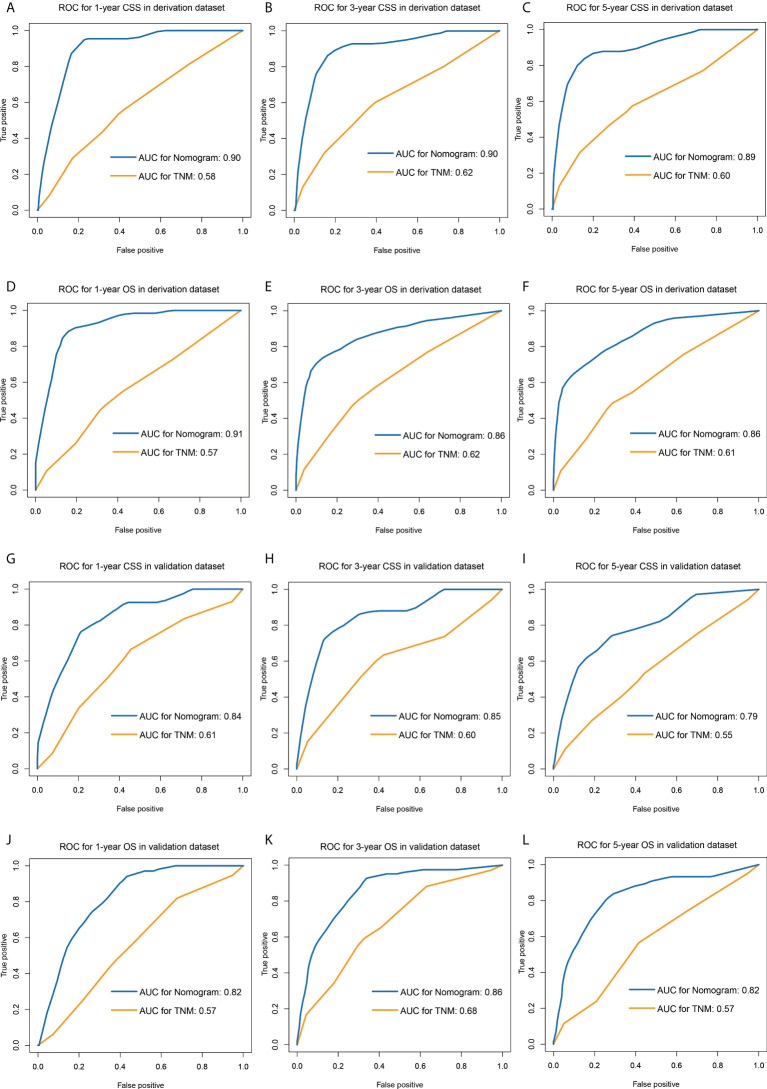
ROC curves of the nomograms and TNM staging system for predicting 1-, 3-, 5-year CSS in the derivation dataset **(A–C)** and external validation dataset **(D–F)**, and 1-, 3-, 5-year OS in the derivation dataset **(G–I)** and external validation dataset **(J–L)**. ROC, receiver operating characteristic; CSS, lung cancer-specific survival; OS, overall survival.

**Figure 5:**
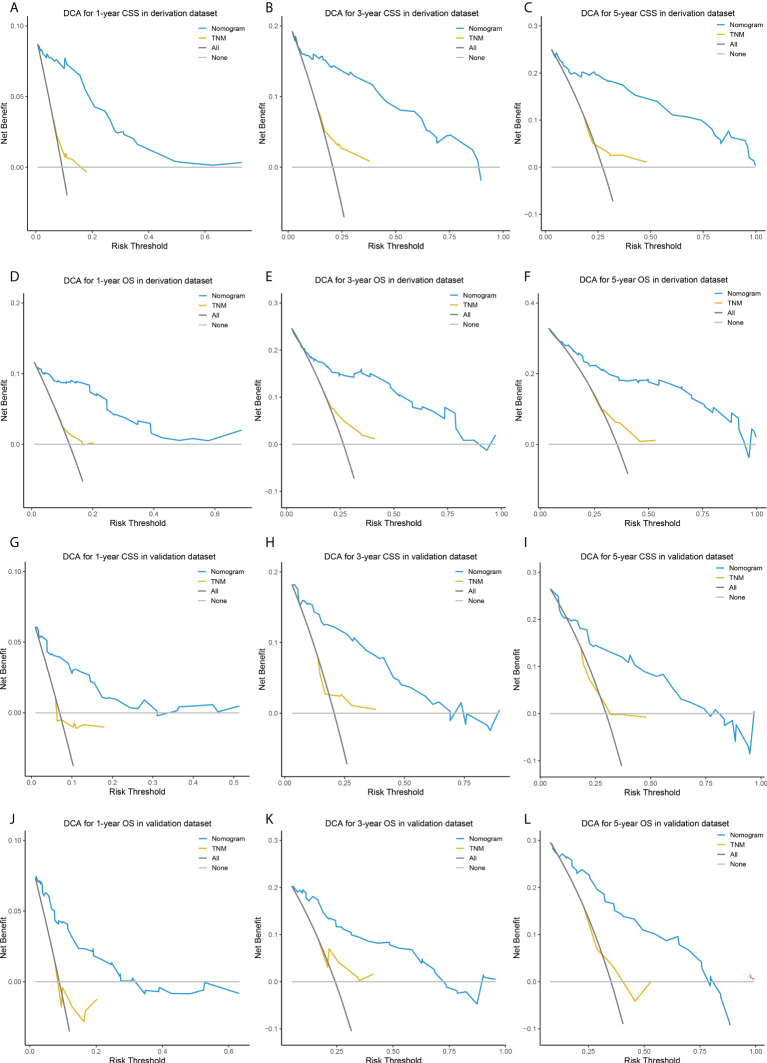
DCA of the nomograms and TNM staging system for predicting 1-, 3-, 5-year CSS in the derivation dataset **(A–C)** and external validation dataset **(D–F)**, and 1-, 3-, 5-year OS in the derivation dataset **(G–I)** and external validation dataset **(J–L)**. DCA, decision curve analysis; CSS, lung cancer-specific survival; OS, overall survival.

**Figure 6:**
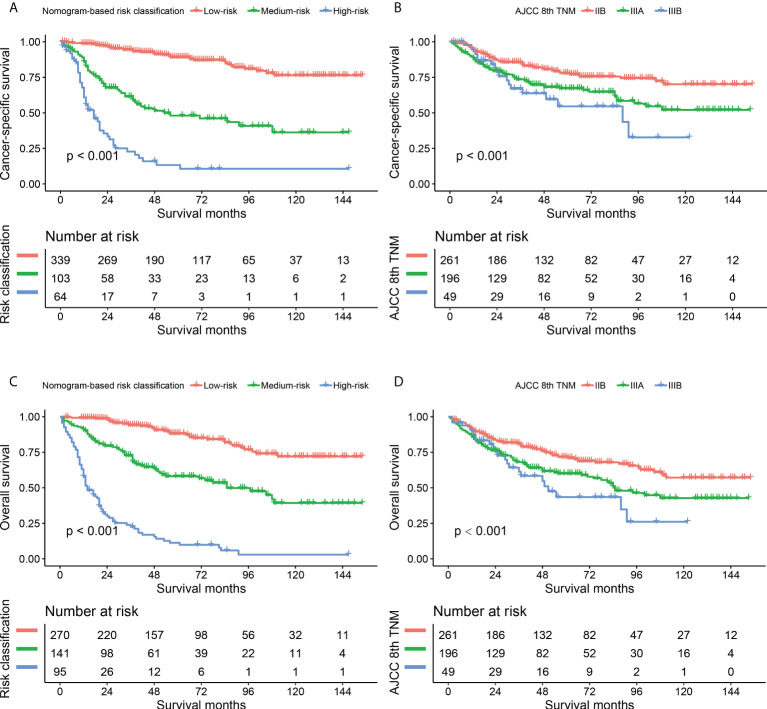
Kaplan-Maier curves comparing nomogram-based classification with 8th AJCC TNM staging system in CSS **(A, B)** and OS **(C, D)** prediction. CSS, cancer-specific survival; OS, overall survival.

The comparisons between the nomograms and the TNM staging system were also performed (**Table S5**). Analysis of accuracy showed that the IDI or NRI for the 1-, 3-, and 5-year CSS or OS in derivation or validation dataset were all larger than 0 with all *P*<0.001, indicating a better prediction power of the nomograms compared with TNM staging system. The area under ROC curve of the nomograms was larger than TNM staging system for the 1-, 3-, and 5-year CSS or OS prediction in derivation or validation dataset (**Figure 4**). Furthermore, the DCA was applied to determine the clinical applicability. The DCA showed that our nomograms were better than the TNM staging system, as it added more net benefits than the TNM classification for nearly all threshold probabilities based on both the derivation cohort and validation cohort (**Figure 5**). The nomogram-calculated total points of the patients were divided into low- (CSS: <80; OS: <83), medium- (CSS: 80 to 133; OS: 83 to 135), and high-risk (CSS: >133; OS: >135) subgroups using X-tile software, and this classification method exhibited better discriminative power than the eighth edition of TNM staging system as shown in the Kaplan-Maier curve analysis (**Figure 6**).

### Establishment of online models for convenient clinical use

Two online nomograms with interactive interface based on the multivariable Cox regression models were built (CSS: https://drboidedwater.shinyapps.io/DynNom-CSS-lungneuroendocrinecarcinoma/; OS: https://drboidedwater.shinyapps.io/DynNom-OS-lungneuroendocrinecarcinoma/). To facilitate the access of these two links, the quick response codes were demonstrated in **Figure 2**. The webservers may generate estimated survival rate and Kaplan-Meier curves by entering the covariates.

## Discussion

LNETs constitute a unique clinical subgroup of primary pulmonary tumors. Due to their relatively low incidence, no specific staging system exists for LNETs. An exact and reasonable classification of the lymph node status is critical in the staging and prognosis evaluation of patients with LNCTs. In this study, LODDS was proved to be independently related to long-term clinical prognosis among patients with resectable LNETs. These results were robust to a series of sensitivity analyses. Second, we constructed two visualized and publicly accessible online nomograms, incorporating LODDS and routinely available demographic, staging and treatment information, to predict the survival probability for individual LNET patients. To our knowledge, the present study is the first to explore the prognostic value of LODDS for LNET based on a multi-center cohort with a relatively large sample size.

The involvement of regional lymph nodes in malignancies has been considered as one of the most important prognostic factors. The 8th version of the TNM staging system of NSCLC divided metastasis to intrapulmonary LNs and ipsilateral peribronchial and/or hilar LNs as N1 classification, and metastasis into ipsilateral mediastinal and/or subcarinal LNs into N2 classification without taking into account numbers of examined and metastatic lymph nodes (46). LODDS is a new LN ratio-based index and has been reported to be as an independent predictor in many malignancies such as rectal cancer (47), pancreatic cancer (48), gallbladder cancer (49), gastric cancer (50), and colon cancer (51). Recently, several studies were attempting to explore the prognostic value of LODDS for NSCLC. In 2020, we did a research and found that the high value of LODDS>-0.37 was independently related to worse CSS/OS in patients with node-positive lung squamous cell cancer (25). Dziedzic et al. (24) found that it is possible to discriminate NSCLC patients more effectively by using LODDS compared to conventional N classification. Deng et al. (23) found LODDS and lymph node ratio (LNR) staging schemes outperformed NPLN for predicting CSS/OS among patients with node-positive NSCLC. However, most previous studies only focused on NSCLC, and few reports have detected the predictive value of LODDS in LNETs.

In the present study, the high value of LODDS was associated with worse survival for N1/N2 stage patients with LNETs. However, LODDS must be used and calculated with caution, because the value of LODDS is influenced by the number of dissected LN. Therefore, sensitivity analyses were performed. For LNETs, the extent of LN management should conform to the International Association for the Study of Lung Cancer (IASLC) recommendations, which suggested examination of at least 6 nodes/stations, 3 of which should be mediastinal including the subcarinal station (52). Considering that the accurate value of LODDS was dependent on the adequate NDLN, we excluded patients with examined LN count less than 6 and found that LODDS could still serve as an independent predictor for LNETs. What's more, it is quite easy to break the integrity of the LN during surgery, especially when the LNs are adherent to one another or challenging to be removed from the dissected tissues (53). To avoid the potential bias led by fragmented LNs, we excluded patients with examined LN count of more than 20, and the results did not change. In summary, LODDS is the ratio-based LN staging system that combines NPLN and NDLN, which might be superior to some number-based LN assessment methods. Furthermore, in LODDS, the numerator and denominator are both added with a value of 0.5, eliminating the singularities caused by null data, therefore LODDS might be used to estimate survival of node-negative patients, as opposed to LNR.

The LODDS was not the only prognostic factor included in our nomograms. Similar to previous studies (54–56), age at diagnosis, sex, histotype, surgery, and radiotherapy were chosen as prognostic factors of CSS or OS. In this study, the nomograms showed that histotype contributed the most to the prognosis, which indicated tumor histotype is a crucial determinant of the clinical behavior of LNETs. Our nomograms indicated that LCNEC showed the worst prognosis followed by AC and TC. Growing evidence also suggests that high-grade LCNEC is biologically distinct from low-grade TC and intermediate-grade AC in view of clinical behavior, pathologic features, molecular alterations as well as possible precursor lesions (57). All-stage 5-year OS for LCNEC fluctuated between 13% and 57% (58). Different from LCNEC, TC and AC are more commonly found in younger patients without smoking histories. AC is significantly more aggressive than TC, with a higher frequency of nodal and distant metastases, and 5-year survival of 60%. In this study, we did not include SCLC patients, because SCLC is usually deemed as nonsurgical (59).

The optimal surgical choice for LNETs is controversial. The surgical type is associated with tumor site, tumor size, and precise assessment of preoperative biopsy specimen. Several studies reported that wedge resection might be correlated with the increased tumor recurrence rate especially in node-positive TC or AC (60, 61). Lobectomy is reported as superior to segmentectomy in terms of OS in some, but not in all pulmonary carcinoids (62–64). Similar to these studies, our study showed that lobectomy was superior to sublobectomy and pneumonectomy in the nomogram for CSS. Furthermore, there is an absence of high-quality evidence to show whether or not chemotherapy could provide clinical benefits for LNETs. Although our nomogram showed that chemotherapy might be associated with more favorable prognoses. However, the results need to be interpreted with caution. In the SEER database, patients without receiving chemotherapy and those with unknown information about adjuvant therapy were classified into one category, which might lead to potential bias. Until now, for pulmonary carcinoids, routine adjuvant therapy may only be considered in selected fit patients (AC, N2 stage) with a particularly high risk of relapse (65). Besides, Iyoda et al. (66) suggested that platinum-based adjuvant chemotherapy after surgery could help patients with LCNEC prevent recurrence.

Because LNET is a heterogeneous disease, each LNET patient requires an individualized and timely risk assessment, which allows for more precise therapeutic strategies and medical resource allocation decisions. In this study, we developed and validated two nomograms to predict prognosis in patients with LNET. Our nomograms based on LODDS were more accurate and obtained more clinical net benefit than the conventional AJCC/UICC TNM staging system. In summary, the online nomograms, composed of several easily obtained predictors, could be a simpler way to engage clinicians in death risks, patient counseling, and decision-making. To put it another way, LNET patients with poorer clinical results estimated by nomograms may require more aggressive therapy (39).

Several limitations of this study should be noted. First, the SEER database lacks some detailed data, such as smoking history, some promising molecular markers (e.g. Ki-67), imaging techniques used before surgery, histological and morphological data (e.g. mitotic rate), type of resection (R0, R1, or R2), and use of systemic therapies. Therefore, they could not be included as covariables in the multivariable Cox models. Second, information about comorbidities, and tumor recurrence is also not available in the database. Third, although we reclassified the TNM classification according to the eighth edition of AJCC/UICC TNM classification, the TNM staging system, which was derived from the SEER database’s collaborative stage system, is a combination of clinical and pathologic stages. Because of the distinction between clinical and pathologic stages, more subgroup analysis is required using a single clinical or pathologic staging system.

## Conclusions

LODDS was found to be useful in predicting CSS/OS in LNET patients who underwent surgery. Webservers of nomograms including LODDS to assess CSS and OS were established. The well-executed nomograms may aid clinicians in providing reasonable, customized therapeutic strategies for LNET patients.

## Data availability statement

The raw data supporting the conclusions of this article will be made available by the authors, without undue reservation.

## Ethics statement

Ethical review and approval was not required for the study on human participants in accordance with the local legislation and institutional requirements. Written informed consent for participation was not required for this study in accordance with the national legislation and the institutional requirements.

## Author contributions

Conception/Design: SW, YY, and ML. Collection and/or assembly of data: JW, YG and ML. Data analysis and interpretation: SW and QX. Manuscript writing: YY, XL, and QX. Final approval of manuscript: All authors. Funding support: XL. All authors contributed to the article and approved the submitted version.

## Funding

This study was funded by Program of Shanghai Academic Research Leader (No. 21XD1402800) and Shanghai “Rising Stars of Medical Talent” Youth Development Program: Outstanding Youth Medical Talents.

## Acknowledgments

The authors would like to thank all patients and staff who have participated in the SEER program.

## Conflict of interest

The authors declare that the research was conducted in the absence of any commercial or financial relationships that could be construed as a potential conflict of interest.

## Publisher’s note

All claims expressed in this article are solely those of the authors and do not necessarily represent those of their affiliated organizations, or those of the publisher, the editors and the reviewers. Any product that may be evaluated in this article, or claim that may be made by its manufacturer, is not guaranteed or endorsed by the publisher.

## Abbreviations

AC, atypical carcinoid; AJCC, American Joint Committee on Cancer; CI, confidence interval; C-index, concordance index; CSS, cancer-specific survival; DCA, decision curve analysis; HR, hazard ratio; IASLC, International Association for the Study of Lung Cancer; IDI, integrated discrimination improvement; IQR, interquartile range; LCNEC, large cell neuroendocrine carcinoma; LN, lymph node; LNET, lung neuroendocrine tumor; LNR, lymph node ratio; LODDS, log odds of positive lymph nodes; NDLN, number of dissected lymph nodes; NPLN, number of positive lymph nodes; NRI, net reclassification improvement; NSCLC, non-small cell lung cancer; OS, overall survival; PRCDA, purchased/referred care delivery area; SCLC, small cell lung carcinoma; SD, standard deviation; SEER, Surveillance, Epidemiology, and End Results; TC, typical carcinoid; TNM, tumor-node-metastasis; UICC, Union for International Cancer Control; WHO, World Health Organization.
