# Supplementary material for: Construction and validation of nomograms based on the log odds of positive lymph nodes to predict the prognosis of lung neuroendocrine tumors
Source: Front Immunol. 2022 Sep 23;13:987881. doi: 10.3389/fimmu.2022.987881 (PMC9539638; doi:10.3389/fimmu.2022.987881)
Supplement: Supplementary file 1 [file DataSheet_1.docx]

**Supplemental tables**

**Table S1**. Univariable and stepwise multivariable Cox proportional regression analysis for the influence of LODDS on CSS of the patients with number of dissected lymph nodes≤20 in derivation cohort

| **Characteristic** | **N (%)** | **Univariable analysis** | |  | **Stepwise multivariable analysis** | |
| --- | --- | --- | --- | --- | --- | --- |
|  |  | **HR (95%CI)** | ***P* value** |  | **HR (95%CI)** | ***P* value** |
| **Age at diagnosis (year)** |  |  |  |  |  |  |
| <65 | 169 (61.9) | 1 |  |  | 1 |  |
| ≥65 | 104 (38.1) | 2.46 (1.55-3.93) | <0.001 |  | 1.86 (1.13-3.06) | 0.014 |
| **Sex** |  |  |  |  |  |  |
| Male | 118 (43.2) | 1 |  |  |  |  |
| Female | 155 (56.8) | 0.68 (0.43-1.08) | 0.099 |  |  |  |
| **Race** |  |  |  |  |  |  |
| White | 256 (93.8) | 1 |  |  |  |  |
| Black | 14 (5.1) | 1.15 (0.42-3.16) | 0.783 |  |  |  |
| Other | 3 (1.1) | 0.00 (0.00-Inf) | 0.996 |  |  |  |
| **Laterality** |  |  |  |  |  |  |
| Right | 145 (53.1) | 1 |  |  |  |  |
| Left | 128 (46.9) | 0.81 (0.51-1.29) | 0.376 |  |  |  |
| **Site** |  |  |  |  |  |  |
| Upper lobe | 116 (42.5) | 1 |  |  |  |  |
| Middle lobe | 32 (11.7) | 0.28 (0.10-0.77) | 0.014 |  |  |  |
| Lower lobe | 107 (39.2) | 0.60 (0.37-0.99) | 0.045 |  |  |  |
| Other | 18 (6.6) | 0.28 (0.07-1.14) | 0.076 |  |  |  |
| **Histotype** |  |  |  |  |  |  |
| Large cell neuroendocrine carcinoma | 76 (27.8) | 1 |  |  | 1 |  |
| Carcinoid tumor | 144 (52.7) | 0.07 (0.04-0.13) | <0.001 |  | 0.06 (0.03-0.11) | <0.001 |
| Atypical carcinoid tumor | 53 (19.4) | 0.16 (0.08-0.32) | <0.001 |  | 0.14 (0.07-0.29) | <0.001 |
| **Differentiation** |  |  |  |  |  |  |
| Well/moderately differentiated | 111 (40.7) | 1 |  |  |  |  |
| Poorly differentiated /undifferentiated | 57 (20.9) | 8.48 (4.50-15.98) | <0.001 |  |  |  |
| Unknown | 105 (38.5) | 1.74 (0.88-3.47) | 0.112 |  |  |  |
| **T classification** |  |  |  |  |  |  |
| T1 | 119 (43.6) | 1 |  |  |  |  |
| T2 | 105 (38.5) | 0.99 (0.59-1.66) | 0.959 |  |  |  |
| T3 | 39 (14.3) | 1.46 (0.74-2.85) | 0.273 |  |  |  |
| T4 | 10 (3.7) | 1.14 (0.35-3.74) | 0.83 |  |  |  |
| **N classification** |  |  |  |  |  |  |
| N1 | 175 (64.1) | 1 |  |  |  |  |
| N2 | 98 (35.9) | 1.72 (1.08-2.74) | 0.021 |  |  |  |
| **Surgery** |  |  |  |  |  |  |
| Sublobectomy | 23 (8.4) | 1 |  |  |  |  |
| Lobectomy | 226 (82.8) | 0.32 (0.17-0.60) | <0.001 |  |  |  |
| Pneumonectomy | 24 (8.8) | 0.39 (0.16-0.96) | 0.041 |  |  |  |
| **Radiotherapy** |  |  |  |  |  |  |
| No/unknown | 235 (86.1) | 1 |  |  |  |  |
| Yes | 38 (13.9) | 2.46 (1.47-4.13) | <0.001 |  |  |  |
| **Chemotherapy** |  |  |  |  |  |  |
| No/unknown | 198 (72.5) | 1 |  |  | 1 |  |
| Yes | 75 (27.5) | 2.85 (1.79-4.53) | <0.001 |  | 0.63 (0.36-1.10) | 0.106 |
| **LODDS as a categorical variable** |  |  |  |  |  |  |
| LODDS<-0.79 | 167 (61.2) | 1 |  |  | 1 |  |
| LODDS≥-0.79 | 106 (38.8) | 2.39 (1.50-3.81) | <0.001 |  | 2.06 (1.28-3.31) | 0.003 |

^†^CSS, cancer-specific survival; LODDS, log odds of positive lymph node; HR, hazard ratio; CI, confidence interval.

**Table S2**. Univariable and sepwise multivariable Cox proportional regression analysis for the influence of LODDS on OS of the patients with number of dissected lymph nodes≤20 in derivation cohort

| **Characteristic** | **N (%)** | **Univariable analysis** | |  | **Stepwise multivariable analysis** | |
| --- | --- | --- | --- | --- | --- | --- |
|  |  | **HR (95%CI)** | ***P* value** |  | **HR (95%CI)** | ***P* value** |
| **Age at diagnosis (year)** |  |  |  |  |  |  |
| <65 | 169 (61.9) | 1 |  |  | 1 |  |
| ≥65 | 104 (38.1) | 3.15 (2.09-4.74) | <0.001 |  | 2.34 (1.52-3.61) | <0.001 |
| **Sex** |  |  |  |  |  |  |
| Male | 118 (43.2) | 1 |  |  |  |  |
| Female | 155 (56.8) | 0.62 (0.42-0.93) | 0.021 |  |  |  |
| **Race** |  |  |  |  |  |  |
| White | 256 (93.8) | 1 |  |  |  |  |
| Black | 14 (5.1) | 1.08 (0.44-2.67) | 0.86 |  |  |  |
| Other | 3 (1.1) | 0.00 (0.00-Inf) | 0.995 |  |  |  |
| **Laterality** |  |  |  |  |  |  |
| Right | 145 (53.1) | 1 |  |  |  |  |
| Left | 128 (46.9) | 0.87 (0.58-1.29) | 0.481 |  |  |  |
| **Site** |  |  |  |  |  |  |
| Upper lobe | 116 (42.5) | 1 |  |  |  |  |
| Middle lobe | 32 (11.7) | 0.40 (0.18-0.88) | 0.022 |  |  |  |
| Lower lobe | 107 (39.2) | 0.73 (0.47-1.12) | 0.149 |  |  |  |
| Other | 18 (6.6) | 0.46 (0.17-1.28) | 0.139 |  |  |  |
| **Histotype** |  |  |  |  |  |  |
| Large cell neuroendocrine carcinoma | 76 (27.8) | 1 |  |  | 1 |  |
| Carcinoid tumor | 144 (52.7) | 0.11 (0.07-0.18) | <0.001 |  | 0.08 (0.04-0.14) | <0.001 |
| Atypical carcinoid tumor | 53 (19.4) | 0.20 (0.11-0.36) | <0.001 |  | 0.16 (0.09-0.31) | <0.001 |
| **Differentiation** |  |  |  |  |  |  |
| Well/moderately differentiated | 111 (40.7) | 1 |  |  |  |  |
| Poorly differentiated /undifferentiated | 57 (20.9) | 7.22 (4.10-12.71) | <0.001 |  |  |  |
| Unknown | 105 (38.5) | 2.18 (1.23-3.88) | 0.008 |  |  |  |
| **T classification** |  |  |  |  |  |  |
| T1 | 119 (43.6) | 1 |  |  |  |  |
| T2 | 105 (38.5) | 1.16 (0.74-1.80) | 0.522 |  |  |  |
| T3 | 39 (14.3) | 1.37 (0.74-2.54) | 0.311 |  |  |  |
| T4 | 10 (3.7) | 1.25 (0.44-3.50) | 0.675 |  |  |  |
| **N classification** |  |  |  |  |  |  |
| N1 | 175 (64.1) | 1 |  |  |  |  |
| N2 | 98 (35.9) | 1.84 (1.23-2.74) | 0.003 |  |  |  |
| **Surgery** |  |  |  |  |  |  |
| Sublobectomy | 23 (8.4) | 1 |  |  |  |  |
| Lobectomy | 226 (82.8) | 0.39 (0.22-0.71) | 0.002 |  |  |  |
| Pneumonectomy | 24 (8.8) | 0.42 (0.18-0.96) | 0.041 |  |  |  |
| **Radiotherapy** |  |  |  |  |  |  |
| No/unknown | 235 (86.1) | 1 |  |  |  |  |
| Yes | 38 (13.9) | 2.07 (1.30-3.32) | 0.002 |  |  |  |
| **Chemotherapy** |  |  |  |  |  |  |
| No/unknown | 198 (72.5) | 1 |  |  | 1 |  |
| Yes | 75 (27.5) | 1.99 (1.32-3.00) | <0.001 |  | 0.53 (0.32-0.86) | 0.011 |
| **LODDS as a categorical variable** |  |  |  |  |  |  |
| LODDS<-0.79 | 167 (61.2) | 1 |  |  | 1 |  |
| LODDS≥-0.79 | 106 (38.8) | 1.89 (1.27-2.83) | 0.002 |  | 1.68 (1.12-2.53) | 0.012 |

^†^OS, overall survival; LODDS, log odds of positive lymph node; HR, hazard ratio; CI, confidence interval.

**Table S3**. Univariable and stepwise multivariable Cox proportional regression analysis for the influence of LODDS on CSS of the patients with number of dissected lymph nodes≥6 in derivation cohort

| **Characteristic** | **N (%)** | **Univariable analysis** | |  | **Stepwise multivariable analysis** | |
| --- | --- | --- | --- | --- | --- | --- |
|  |  | **HR (95%CI)** | ***P* value** |  | **HR (95%CI)** | ***P* value** |
| **Age at diagnosis (year)** |  |  |  |  |  |  |
| <65 | 141 (62.4) | 1 |  |  | 1 |  |
| ≥65 | 85 (37.6) | 2.32 (1.35-3.99) | 0.002 |  | 1.70 (0.96-3.01) | 0.067 |
| **Sex** |  |  |  |  |  |  |
| Male | 96 (42.5) | 1 |  |  |  |  |
| Female | 130 (57.5) | 0.78 (0.45-1.34) | 0.366 |  |  |  |
| **Race** |  |  |  |  |  |  |
| White | 211 (93.4) | 1 |  |  |  |  |
| Black | 11 (4.9) | 1.07 (0.33-3.44) | 0.907 |  |  |  |
| Other | 4 (1.8) | 0.00 (0.00-Inf) | 0.997 |  |  |  |
| **Laterality** |  |  |  |  |  |  |
| Right | 121 (53.5) | 1 |  |  |  |  |
| Left | 105 (46.5) | 1.04 (0.61-1.79) | 0.877 |  |  |  |
| **Site** |  |  |  |  |  |  |
| Upper lobe | 86 (38.1) | 1 |  |  |  |  |
| Middle lobe | 25 (11.1) | 0.27 (0.08-0.88) | 0.03 |  |  |  |
| Lower lobe | 88 (38.9) | 0.53 (0.29-0.97) | 0.038 |  |  |  |
| Other | 27 (11.9) | 0.49 (0.19-1.26) | 0.138 |  |  |  |
| **Histotype** |  |  |  |  |  |  |
| Large cell neuroendocrine carcinoma | 60 (26.5) | 1 |  |  | 1 |  |
| Carcinoid tumor | 117 (51.8) | 0.10 (0.05-0.19) | <0.001 |  | 0.07 (0.03-0.15) | <0.001 |
| Atypical carcinoid tumor | 49 (21.7) | 0.16 (0.07-0.36) | <0.001 |  | 0.11 (0.05-0.27) | <0.001 |
| **Differentiation** |  |  |  |  |  |  |
| Well/moderately differentiated | 90 (39.8) | 1 |  |  |  |  |
| Poorly differentiated /undifferentiated | 47 (20.8) | 6.44 (3.18-13.07) | <0.001 |  |  |  |
| Unknown | 89 (39.4) | 1.22 (0.57-2.65) | 0.606 |  |  |  |
| **T classification** |  |  |  |  |  |  |
| T1 | 89 (39.4) | 1 |  |  | 1 |  |
| T2 | 91 (40.3) | 0.69 (0.37-1.29) | 0.242 |  | 0.52 (0.27-1.01) | 0.053 |
| T3 | 34 (15.0) | 1.18 (0.54-2.55) | 0.677 |  | 1.17 (0.53-2.58) | 0.697 |
| T4 | 12 (5.3) | 1.23 (0.43-3.58) | 0.7 |  | 1.45 (0.42-5.01) | 0.558 |
| **N classification** |  |  |  |  |  |  |
| N1 | 149 (65.9) | 1 |  |  |  |  |
| N2 | 77 (34.1) | 1.26 (0.73-2.19) | 0.404 |  |  |  |
| **Surgery** |  |  |  |  |  |  |
| Sublobectomy | 11 (4.9) | 1 |  |  | 1 |  |
| Lobectomy | 188 (83.2) | 0.32 (0.12-0.81) | 0.016 |  | 0.18 (0.06-0.49) | <0.001 |
| Pneumonectomy | 27 (11.9) | 0.57 (0.19-1.65) | 0.298 |  | 0.43 (0.13-1.40) | 0.161 |
| **Radiotherapy** |  |  |  |  |  |  |
| No/unknown | 201 (88.9) | 1 |  |  |  |  |
| Yes | 25 (11.1) | 2.64 (1.41-4.94) | 0.002 |  |  |  |
| **Chemotherapy** |  |  |  |  |  |  |
| No/unknown | 165 (73.0) | 1 |  |  |  |  |
| Yes | 61 (27.0) | 3.16 (1.84-5.42) | <0.001 |  |  |  |
| **LODDS as a categorical variable** |  |  |  |  |  |  |
| LODDS<-0.79 | 170 (75.2) | 1 |  |  | 1 |  |
| LODDS≥-0.79 | 56 (24.8) | 2.24 (1.28-3.91) | 0.005 |  | 3.64 (1.99-6.68) | <0.001 |

^†^CSS, cancer-specific survival; LODDS, log odds of positive lymph node; HR, hazard ratio; CI, confidence interval.

**Table S4**. Univariable and stepwise multivariable Cox proportional regression analysis for the influence of LODDS on OS of the patients with number of dissected lymph nodes≥6 in derivation cohort

| **Characteristic** | **N (%)** | **Univariable analysis** | |  | **Stepwise multivariable analysis** | |
| --- | --- | --- | --- | --- | --- | --- |
|  |  | **HR (95%CI)** | ***P* value** |  | **HR (95%CI)** | ***P* value** |
| **Age at diagnosis (year)** |  |  |  |  |  |  |
| <65 | 141 (62.4) | 1 |  |  | 1 |  |
| ≥65 | 85 (37.6) | 3.03 (1.94-4.73) | <0.001 |  | 2.30 (1.45-3.64) | <0.001 |
| **Sex** |  |  |  |  |  |  |
| Male | 96 (42.5) | 1 |  |  |  |  |
| Female | 130 (57.5) | 0.65 (0.42-1.00) | 0.051 |  |  |  |
| **Race** |  |  |  |  |  |  |
| White | 211 (93.4) | 1 |  |  |  |  |
| Black | 11 (4.9) | 1.20 (0.48-2.96) | 0.695 |  |  |  |
| Other | 4 (1.8) | 0.00 (0.00-Inf) | 0.996 |  |  |  |
| **Laterality** |  |  |  |  |  |  |
| Right | 121 (53.5) | 1 |  |  |  |  |
| Left | 105 (46.5) | 0.95 (0.61-1.47) | 0.803 |  |  |  |
| **Site** |  |  |  |  |  |  |
| Upper lobe | 86 (38.1) | 1 |  |  |  |  |
| Middle lobe | 25 (11.1) | 0.51 (0.22-1.14) | 0.102 |  |  |  |
| Lower lobe | 88 (38.9) | 0.75 (0.46-1.24) | 0.261 |  |  |  |
| Other | 27 (11.9) | 0.82 (0.41-1.67) | 0.585 |  |  |  |
| **Histotype** |  |  |  |  |  |  |
| Large cell neuroendocrine carcinoma | 60 (26.5) | 1 |  |  | 1 |  |
| Carcinoid tumor | 117 (51.8) | 0.15 (0.09-0.25) | <0.001 |  | 0.10 (0.05-0.19) | <0.001 |
| Atypical carcinoid tumor | 49 (21.7) | 0.20 (0.10-0.39) | <0.001 |  | 0.15 (0.07-0.31) | <0.001 |
| **Differentiation** |  |  |  |  |  |  |
| Well/moderately differentiated | 90 (39.8) | 1 |  |  |  |  |
| Poorly differentiated /undifferentiated | 47 (20.8) | 5.77 (3.13-10.64) | <0.001 |  |  |  |
| Unknown | 89 (39.4) | 1.75 (0.95-3.25) | 0.074 |  |  |  |
| **T classification** |  |  |  |  |  |  |
| T1 | 89 (39.4) | 1 |  |  |  |  |
| T2 | 91 (40.3) | 0.93 (0.57-1.52) | 0.766 |  |  |  |
| T3 | 34 (15.0) | 1.05 (0.53-2.09) | 0.88 |  |  |  |
| T4 | 12 (5.3) | 1.14 (0.44-2.94) | 0.783 |  |  |  |
| **N classification** |  |  |  |  |  |  |
| N1 | 149 (65.9) | 1 |  |  |  |  |
| N2 | 77 (34.1) | 1.48 (0.95-2.31) | 0.08 |  |  |  |
| **Surgery** |  |  |  |  |  |  |
| Sublobectomy | 11 (4.9) | 1 |  |  |  |  |
| Lobectomy | 188 (83.2) | 0.50 (0.20-1.25) | 0.137 |  |  |  |
| Pneumonectomy | 27 (11.9) | 0.68 (0.24-1.90) | 0.458 |  |  |  |
| **Radiotherapy** |  |  |  |  |  |  |
| No/unknown | 201 (88.9) | 1 |  |  |  |  |
| Yes | 25 (11.1) | 1.93 (1.10-3.38) | 0.023 |  |  |  |
| **Chemotherapy** |  |  |  |  |  |  |
| No/unknown | 165 (73.0) | 1 |  |  | 1 |  |
| Yes | 61 (27.0) | 1.95 (1.24-3.07) | 0.004 |  | 0.51 (0.29-0.90) | 0.02 |
| **LODDS as a categorical variable** |  |  |  |  |  |  |
| LODDS<-0.79 | 170 (75.2) | 1 |  |  | 1 |  |
| LODDS≥-0.79 | 56 (24.8) | 1.79 (1.12-2.86) | 0.015 |  | 2.00 (1.23-3.23) | 0.005 |

^†^OS, overall survival; LODDS, log odds of positive lymph node; HR, hazard ratio; CI, confidence interval.

**Table S5**. The IDI and continuous-NRI comparing nomogram with AJCC 8th TNM staging system

| **Endpoint** | **IDI** | |  | **Continuous-NRI** | |
| --- | --- | --- | --- | --- | --- |
|  | **95%CI** | ***P* value** |  | **95%CI** | ***P* value** |
| **Derivation dataset** |  |  |  |  |  |
| 1-year CSS | 0.25 (0.16-0.39) | <0.001 |  | 0.73 (0.56-0.82) | <0.001 |
| 3-year CSS | 0.38 (0.24-0.5) | <0.001 |  | 0.65 (0.42-0.75) | <0.001 |
| 5-year CSS | 0.42 (0.27-0.54) | <0.001 |  | 0.66 (0.43-0.77) | <0.001 |
| 1-year OS | 0.27 (0.18-0.38) | <0.001 |  | 0.68 (0.45-0.79) | <0.001 |
| 3-year OS | 0.35 (0.21-0.46) | <0.001 |  | 0.49 (0.35-0.64) | <0.001 |
| 5-year OS | 0.37 (0.23-0.46) | <0.001 |  | 0.56 (0.36-0.66) | <0.001 |
| **External validation dataset** |  |  |  |  |  |
| 1-year CSS | 0.12 (0.04-0.23) | <0.001 |  | 0.47 (0.21-0.67) | <0.001 |
| 3-year CSS | 0.26 (0.1-0.39) | <0.001 |  | 0.57 (0.31-0.71) | <0.001 |
| 5-year CSS | 0.28 (0.11-0.4) | <0.001 |  | 0.52 (0.28-0.64) | <0.001 |
| 1-year OS | 0.11 (0.04-0.21) | <0.001 |  | 0.43 (0.15-0.62) | <0.001 |
| 3-year OS | 0.25 (0.13-0.37) | <0.001 |  | 0.43 (0.29-0.63) | <0.001 |
| 5-year OS | 0.28 (0.15-0.36) | <0.001 |  | 0.48 (0.31-0.63) | <0.001 |

^†^CSS, cancer-specific survival; IDI, integrated discrimination improvement; LODDS, log odds of positive lymph node; NRI, net reclassification improvement; OS, overall survival.
